# Spatial organization of endometrial gene expression at the onset of embryo attachment in pigs

**DOI:** 10.1186/s12864-019-6264-2

**Published:** 2019-11-21

**Authors:** Shuqin Zeng, Susanne E. Ulbrich, Stefan Bauersachs

**Affiliations:** 1 Genetics and Functional Genomics, Clinic of Reproductive Medicine, Department for Farm Animals, Vetsuisse Faculty, University of Zurich, Eschikon 27 AgroVet-Strickhof, Zurich, Switzerland; 2 Animal Physiology, Institute of Agricultural Sciences, ETH Zurich, Lindau, ZH 8315 Switzerland

**Keywords:** Pig, Preimplantation, Endometrium, Cell type-specific, Transcriptomics, RNA-seq, LMD, LCM

## Abstract

**Background:**

During the preimplantation phase in the pig, the conceptus trophoblast elongates into a filamentous form and secretes estrogens, interleukin 1 beta 2, interferons, and other signaling molecules before attaching to the uterine epithelium. The processes in the uterine endometrium in response to conceptus signaling are complex. Thus, the objective of this study was to characterize transcriptome changes in porcine endometrium during the time of conceptus attachment considering the specific localization in different endometrial cell types.

**Results:**

Low-input RNA-sequencing was conducted for the main endometrial compartments, luminal epithelium (LE), glandular epithelium (GE), blood vessels (BV), and stroma. Samples were isolated from endometria collected on Day 14 of pregnancy and the estrous cycle (each group *n* = 4) by laser capture microdissection. The expression of 12,000, 11,903, 11,094, and 11,933 genes was detectable in LE, GE, BV, and stroma, respectively. Differential expression analysis was performed between the pregnant and cyclic group for each cell type as well as for a corresponding dataset for complete endometrium tissue samples. The highest number of differentially expressed genes (DEGs) was found for LE (1410) compared to GE, BV, and stroma (800, 1216, and 384). For the complete tissue, 3262 DEGs were obtained. The DEGs were assigned to Gene Ontology (GO) terms to find overrepresented functional categories and pathways specific for the individual endometrial compartments. GO classification revealed that DEGs in LE were involved in ‘biosynthetic processes’, ‘related to ion transport’, and ‘apoptotic processes’, whereas ‘cell migration’, ‘cell growth’, ‘signaling’, and ‘metabolic/biosynthetic processes’ categories were enriched for GE. For blood vessels, categories such as ‘focal adhesion’, ‘actin cytoskeleton’, ‘cell junction’, ‘cell differentiation and development’ were found as overrepresented, while for stromal samples, most DEGs were assigned to ‘extracellular matrix’, ‘gap junction’, and ‘ER to Golgi vesicles’.

**Conclusions:**

The localization of differential gene expression to different endometrial cell types provided a significantly improved view on the regulation of biological processes involved in conceptus implantation, such as the control of uterine fluid secretion, trophoblast attachment, growth regulation by Wnt signaling and other signaling pathways, as well as the modulation of the maternal immune system.

## Background

The preimplantation period in the pig involves comprehensive biological events including maternal recognition of pregnancy and preparation for conceptus implantation [1]. Many aspects and regulations at the gene expression level are different and specific compared to other species [2–4]. The intensive molecular crosstalk between implanting embryos and the receptive uterus is a prerequisite to establish a successful pregnancy [5]. After a rapid initial transition of porcine blastocysts from spherical to tubular and elongated filamentous forms between Days 10 and 12 of pregnancy [6], the initial attachment of conceptus trophectoderm to the uterine epithelium starts on approximately Day 13, followed by more stable adhesion observed on Day 16 [7]. On Days 13 and 14, protruding epithelial proliferations of the endometrium enclosed by chorionic caps, immobilize the blastocyst and keep the maternal and fetal sides together to develop cell-cell contacts for a close apposition between the apical plasma membranes of trophoblast and uterine epithelium [8]. Within the attachment sites, the surface area is increased by the presence of endometrial folds, surface epithelial folds, and microvilli between the trophoblast and dome-shaped luminal epithelium (LE) cells that are coated by a thick glycocalyx [7, 8]. Several primary molecules, such as mucins, integrins and CDs, have been shown in regulation of various cell adhesion cascades for the embryo implantation in pigs [9–12]. Among the adhesion molecules, integrin family members serve as receptors for various extracellular matrix (ECM) ligands. They do not only modulate cell-cell adhesion, but are also involved in serial complex signal transduction events [13]. Osteopontin (OPN; also known as SPP1) is a secreted ECM protein that can bind with various integrins on the cell surface, and SPP1 has been identified as a candidate adhesion molecule for implantation in pigs and sheep [14]. A further study has confirmed that SPP1 could directly bind with specific integrins on porcine trophectoderm cells and uterine luminal epithelial cells to promote trophectoderm cell migration and adhesion [15]. A related study about ITGAV in porcine trophoblast showed that ITGAV-containing integrin receptors adhere to SPP1, suggesting that mechanical forces generated by elongating conceptuses to uterine LE leads to the assembly of focal adhesions involving ITGAV and SPP1 [10].

Uterine endometrial receptivity and preparation for implantation takes place along with conceptus development in response to a variety of conceptus signals such as estrogens, interleukin 1 beta 2 (IL1B2), and interferons (IFNs) which is crucial for successful establishment of pregnancy [16]. Until recently, the model of MRP in the pig was that estrogen (E2) produced from the porcine conceptus between Days 11 and 13 regulates nutrients and prostaglandin F2-alpha (PGF) secretion into the uterine lumen rather than into the uterine vein, which results in extension of the corpora lutea (CL) life cycle to facilitate pregnancy recognition [17]. However, a recent study showed that the estrogen signal is not essential for initial MRP and prevention of luteolysis but for maintaining pregnancy after day 25 [18]. The complex interactions between the conceptus and the endometrium required to maintain pregnancy have been investigated in a variety of studies. For example, Franczak et al. reported that cell adhesion molecules and the steroid hormone biosynthesis pathway were the most significantly enriched biological pathways in porcine endometrium on Days 15 to 16 of pregnancy [19]. In the first transcriptome study of porcine endometrium at the beginning of implantation (Day 14), a number of 263 differentially expressed genes (DEGs) were identified in the endometrium of pregnant versus non-pregnant sows at the time of initial placentation, and most of the upregulated genes were involved in functional categories, such as “developmental process”, “transporter activity”, “calcium ion binding”, “apoptosis”, and “cell motility” [20]. In addition to microarray studies based on nucleic acid hybridization, transcriptome changes during the preimplantation phase have been studied by using RNA-seq in our own and other laboratories [21–24], and these studies revealed a variety of processes and molecular pathways potentially involved in the regulation of the endometrial functions during conceptus attachment and implantation. However, the knowledge of cell-specific gene expression in the complex endometrial tissue is still poor and clearly limiting the value of the results of endometrial gene expression studies. Our recent study on Day 12 of pregnancy, the time of initial maternal recognition of pregnancy in the pig revealed complex and very specific localization of endometrial transcriptome changes and many DEGs not detectable as differentially expressed in the analysis of complete tissue samples [25]. On Day 12, the main response with respect to gene expression changes was localized to the luminal epithelium [25]. Furthermore, similar studies of the endometrium in other species also found very cell type-specific localization of differential expression (DE) [26–28]. With the same approach, we aimed here to reveal the endometrial molecular changes at the beginning of the conceptus attachment period on Day 14 in comparison of samples collected from pregnant and cyclic pigs. To reflect the complexity of the endometrial tissue, the four main compartments with different functions, luminal epithelium (LE), glandular epithelium (GE), stromal areas (S), and blood vessels (BV) were studied by laser capture microdissection. All four compartments are considered as important. Regarding their localization, the LE is in first layer, in direct contact to the conceptus and its secretions. The GE is important for the secretion of nutrients and factors important for conceptus growth and development. Blood vessels undergo remodeling during the implantation process (increased vascularization at implantation zones) as well as stromal areas., the latter containing also a variety of important immune cells.

## Results

### Numbers of detectable and differentially expressed genes in LCM samples and complete endometrial tissue samples

Around 500 million raw reads from the LE, GE, BV, and S samples (in total 32 samples) were obtained with RNA-seq, 251 and 249 million reads in pregnant and cyclic groups, respectively. After removal of low quality reads and PCR duplicates, 397 million clean reads (192 million reads in pregnant and 205 million reads in cyclic group) were obtained and used for further analyses in EdgeR [29]. The detailed information of the raw data for each library is shown in Additional file 4: Table S1.

**Fig. 1:**
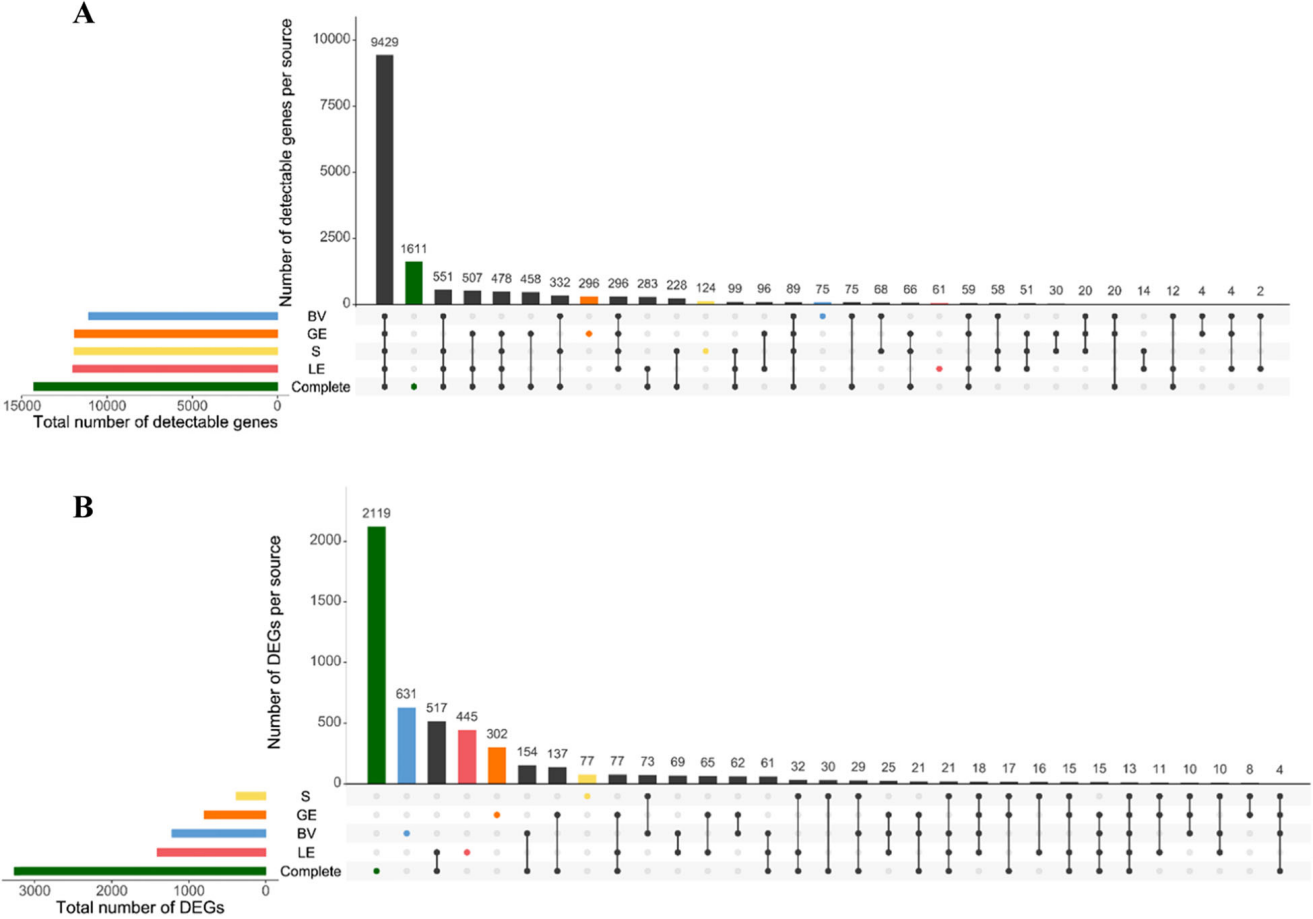
Numbers and overlaps of detectable genes (**a**) and differentially expressed genes (DEGs) (**b**) for the 4 LCM sample types and complete tissue samples illustrated using Upset plots. On the left side, the total numbers of detectable genes and the DEGs, respectively, are shown for complete tissue samples (green), luminal epithelium (red, LE), stromal cells (yellow, S), glandular epithelium (orange, GE), and blood vessel (blue, BV). The colored dots indicate the number of genes specifically detectable (**a**) or specific DEGs (**b**) for the corresponding sample type. Numbers with black dots show the numbers of genes commonly expressed (**a**) or differential (**b**) in different sample types

A number of 12,000, 11,903, 11,094, and 11,933 genes were detectable in LE, GE, BV, and S, respectively (Additional file 5: Table S2). Combining the detected genes from the 4 individual endometrial compartments resulted in a total of 13,885 detected genes. RNA-sequencing of complete endometrial tissue samples revealed slightly more detectable genes (14297). The comparison of LCM samples and complete endometrium showed that the majority of the detectable genes (9429) could be identified in all four individual cell types as well as in the complete tissue (Upset plot, Fig. 1a). In total, 1199 genes were found as expressed in either one or more of the LCM samples but not in the complete tissue sample. A number of 61, 296, 75, and 124 genes were specifically found in LE, GE, BV, and S, respectively.

Comparison of RNA-seq data between pregnant gilts and cyclic controls was used to define DEGs in the current study. The number of DEGs in LCM samples were 1410, 800, 1216, and 384 (LE, GE, BV, and S, respectively; FDR (1%) or corresponding *P* value (0.0012), whereas 3262 DEGs were found in complete endometrial tissue (Additional file 6: Table S3 and Additional file 1: Figure S1, S2, S3, S4). Though a large number of genes were differently expressed (DE) among these cell types, it was notable that only a small number of DEGs (13) were found in all four LCM samples and complete endometrium as differentially expressed, and 18 in all four LCM cell types (Fig. 1b). Besides, 2119 DEGs were only identified in complete endometrium, and 445, 302, 631, and 77 DEGs were specifically obtained for LE, GE, BV, and S, respectively. This points to a highly specific spatial regulation of gene expression. The DE analysis was in addition to EdgeR performed using the tool DESeq2 [30] that revealed very similar lists of DEGs (see Additional file 2: Figure S5 for DEGs complete endometrium).

### Comparison of LCM RNA-seq results to previous data from real-time RT-PCR

Validation of 14 selected genes from complete tissue samples was performed recently using quantitative PCR (dataset from Samborski et al. [22]). The selection of these genes was based on the previous findings of known or inferred functions in the porcine endometrium on Day 14 of pregnancy. The results for these genes were compared with RNA-seq results from the current study using the LCM method. Similar mRNA expression profiles were observed in this comparison (Table 1).

**Table 1:** Comparison of RNA-seq and qPCR data

Ssc gene symbol	Ssc Entrez gene ID	Hsa *gene symbol*	Hsa Entrez gene ID	Hsa gene description	log2 FC P/C						FDR (RNA-seq) / *P*-value (qPCR)					
					RNA-seq					qPCR	RNA-seq					qPCR
					LE	GE	BV	S	Complete		LE	GE	BV	S	Complete	
*DEFB1*	404699	*DEFB1*	1672	defensin beta 1	−4.1				−3.9	−3.4	0.000				0.000	0.010
*FGF9*	396717	*FGF9*	2254	fibroblast growth factor 9	3.4	2.0			2.6	3.0	0.000	0.000			0.000	< 0.001
*IRF1*	396611	*IRF1*	3659	interferon regulatory factor 1	0.8	1.9	0.7	1.8	3.3	2.9	0.045	0.000	0.023	0.000	0.000	0.003
*PLP1*	397029	*PLP1*	5354	proteolipid protein 1					8.3	7.5					0.000	< 0.001
*S100A9*	100127489	*S100A9*	6280	S100 calcium binding protein A9	13.4			8.9	9.1	9.2	0.000			0.000	0.000	< 0.001
*SERPINB7*	100152588	*SERPINB7*	8710	serpin family B member 7	6.3			6.7	4.5	5.4	0.000			0.000	0.000	0.018
*SPP1*	397087	*SPP1*	6696	secreted phosphoprotein 1	0.0	1.0	−1.3	−1.1	3.6	3.2	0.987	0.036	0.002	0.017	0.000	0.029
*STAT1*	396655	*STAT1*	6772	signal transducer and activator of transcription 1	0.7	0.8	0.2	1.0	2.7	2.3	0.029	0.085	0.432	0.008	0.000	< 0.001
*CLDN10*	100153752	*CLDN10*	9071	claudin 10		−0.5			−0.6	−0.5		0.196			0.009	0.089
*CLDN11*	100302016	*CLDN11*	5010	claudin 11				1.6	0.6	0.1				0.000	0.084	0.506
*HPGD*	100156186	*HPGD*	3248	15-hydroxyprostaglandin dehydrogenase	−2.7	4.2	0.4	−1.0	−0.9	−1.3	0.000	0.000	0.241	0.015	0.000	0.055
*LOC102161418*	102161418	*IFITM1*	8519	interferon-induced transmembrane protein 1-like	−1.2		1.0	0.2	−0.6	0.4	0.006		0.005	0.675	0.007	0.195
*PAQR5*	100521597	*PAQR5*	54852	progestin and adipoQ receptor family member 5	0.9	1.8			0.4	0.3	0.073	0.000			0.072	0.195
*STC1*	100125345	*STC1*	6781	stanniocalcin 1	4.2			3.0	2.1	2.0	0.000			0.000	0.000	0.017

### Unsupervised clustering of RNA-seq data sets of the LCM samples

**Fig. 2:**
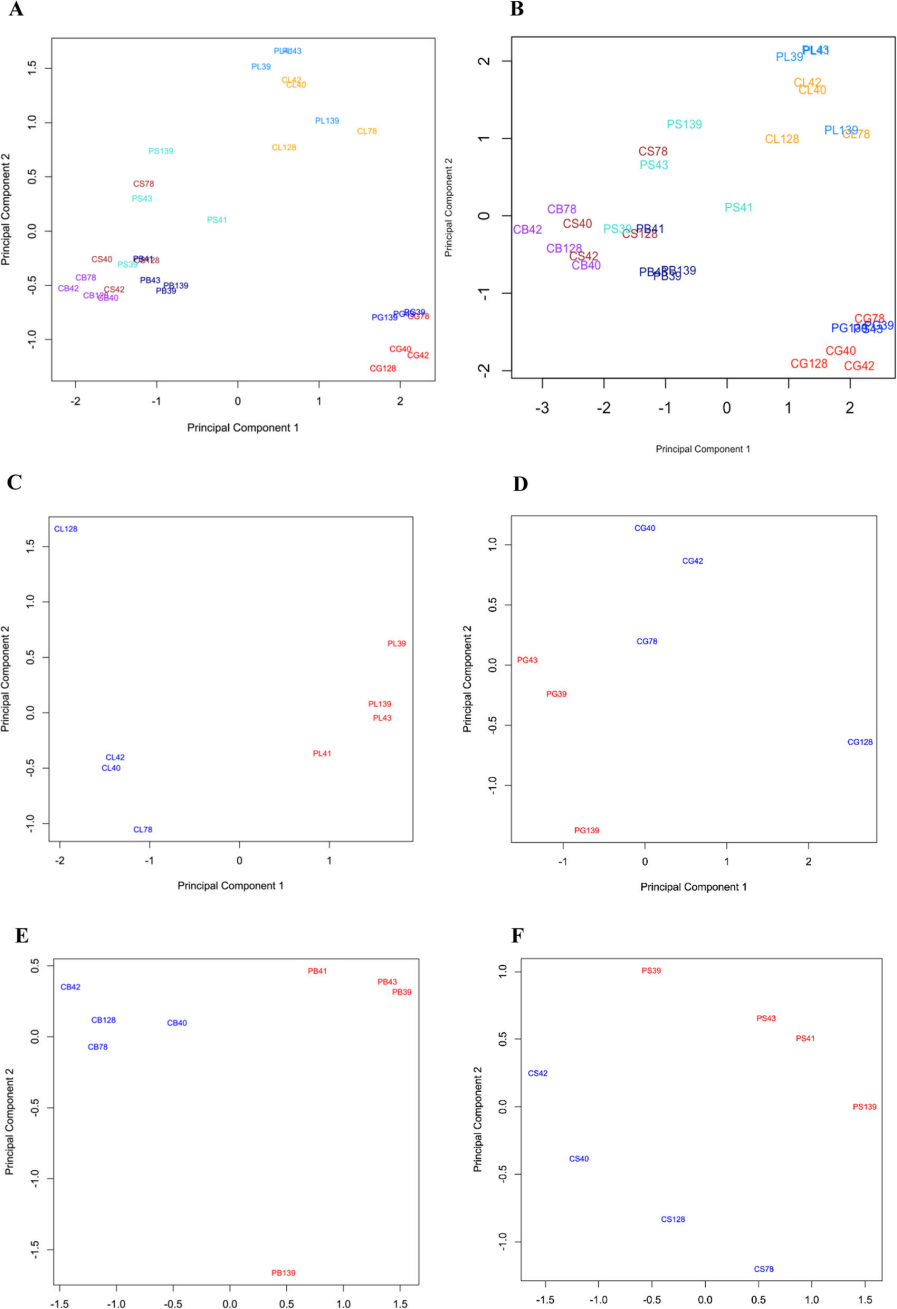
Unsupervised clustering of endometrial LCM samples. Multidimensional scaling plots were generated in EdgeR for the genes showing the highest leading log-fold-changes between the samples in the dataset for LCM samples. Sample groups: CL (orange): cyclic, luminal epithelium; PL (dodgerblue): pregnant, luminal epithelium; CG (red): cyclic, glandular epithelium; PG (blue): pregnant, glandular epithelium; CB (purple): cyclic, blood vessels; PB (darkblue): pregnant, blood vessels; CS (brown): cyclic, stroma; PS (cyan): pregnant, stroma. **a,b** all LCM samples based on the 2000 genes with highest leading log-fold-changes (**a**) and on all detectable genes (**b**). **c** luminal epithelium samples. **d** glandular epithelium samples. **e** blood vessel samples. **f** stroma samples. **c-f** MDS plots based on the 500 genes with highest leading log-fold-changes. Red and Blue indicate samples from pregnant and cyclic groups, respectively

To explore the RNA-seq data in an unsupervised manner, multiple dimension scaling (MDS) plots were generated which are based on leading log-fold-changes between each pair of RNA-seq samples (Fig. 2). In the MDS plot including all LCM samples, a clustering of samples derived from the same cell type including pregnant and cyclic groups was observed for LE, GE, BV, and S (Fig. 2a, b). However, a clear separation of pregnant and control samples was mainly found for BV according to principal component 1. Since the overlap of DEGs in comparison of the different LCM sample types was low, individual MDS plots were also generated for each LCM sample type (Fig. 2c, d, e and f). In the latter MDS plots, a clear separation of samples derived from the pregnant group and the control group was obtained.

In addition, a hierarchical cluster analysis was performed for each individual LCM sample type to show homogeneity of gene expression in the individual samples (biological replicates) of the pregnant and cyclic stage, respectively (see Additional file 1: Figure S1, S2, S3, and S4). Regarding the comparison between pregnant and cyclic endometrium, 833, 501, 643, and 245 DEGs were upregulated in LE, GE, BV, and S of pregnant gilts, respectively, and 577, 299, 573, and 139 DEGs were identified as downregulated in LE, GE, BV, and S, respectively. The detailed information for the obtained DEGs can be found in Additional file 6: Table S3.

### Comparative functional annotation of DEGs between cell types

**Fig. 3:**
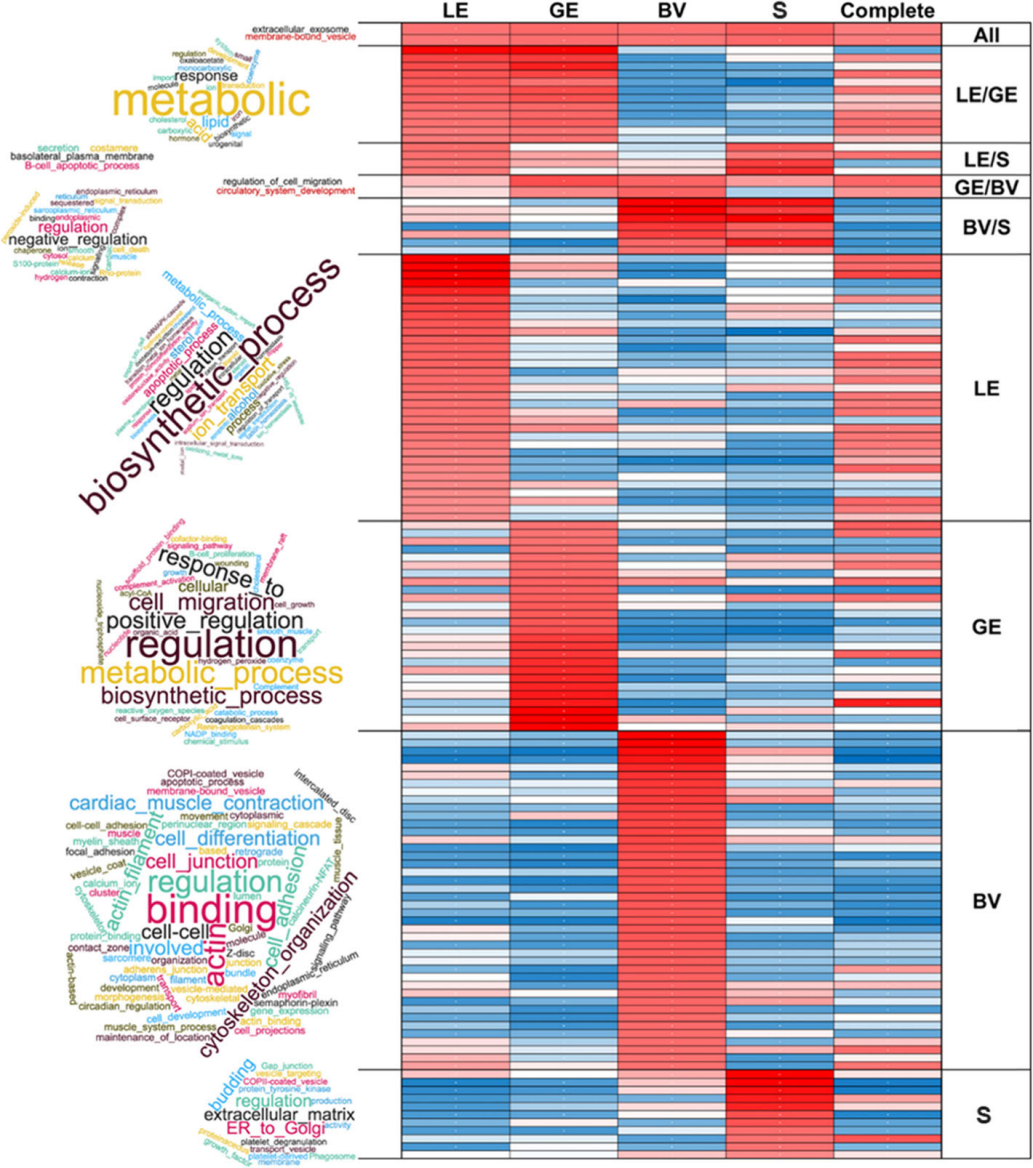
Comparative DAVID Gene Ontology chart analysis. Overrepresentation of the most significantly overrepresented functional categories of each LCM sample type (LE: luminal epithelium, GE: glandular epithelium, BV: blood vessel, S: stroma, All: overrepresented in all sample types) was compared. Categories were filtered manually for redundancy. The word clouds on the left side indicate the main functional categories/terms for the DEGs obtained for the respective endometrial compartments. Characteristic terms and words of the overrepresented categories were used to generate word clouds where the font size indicates the frequency of the word or term. The heatmap shows a score combining fold enrichment and false discovery rate (blue = lowest score, red = score of 7 or higher). For details of the DAVID GO chart analysis see Additional file 7: Table S4

To compare in more detail the cell-specific differential gene expression, functional classification was conducted using the online tool DAVID GO charts (Gene Ontology (GO) categories and KEGG pathways) for the upregulated genes. The functional categories with FDR < 5% were selected, then sorted by a score combining FDR and fold enrichment, and 20% best scores were used for the heatmap and word clouds based on the overrepresented terms and pathways. The results shown in Fig. 3 revealed ‘extracellular exosome’ and ‘membrane bound vesicle’ categories as overrepresented in all four cell types as well as in complete endometrial tissue. For LE and GE, mainly lipid metabolic processes were overrepresented, while secretion, basolateral plasma membrane, and B cell apoptotic process were enriched for LE and stroma. The processes ‘regulation of cell migration’ and ‘circulatory system development’ were obtained for GE and BV. Categories related to regulation of different processes, endoplasmic reticulum were found for BV and stroma. In addition to the commonly enriched functional categories, some GO terms and pathways were specifically enriched for the specific cell types, such as categories describing biosynthetic processes, related to ion transport, and apoptotic processes were enriched for the genes upregulated in LE. In contrast, overrepresented categories and pathways in GE were related to cell migration, cell growth, signaling, and metabolic/biosynthetic processes. Functional categories and pathways such as ‘focal adhesion’, ‘actin cytoskeleton’, ‘cell junction’, ‘cell differentiation and development’ were highly enriched for BV. For stroma, genes related to extracellular matrix, gap junction, and ER to Golgi vesicles were overrepresented. The detailed information can be found in Additional file 7: Table S4. Among all these functional categories and pathways, it is of notice that overrepresentation of adhesion functions was most significant for genes upregulated in BV, and for all cell types various cell communication categories were found as overrepresented.

### Top 20 DEGs of LCM samples and complete endometrial tissue

**Fig. 4:**
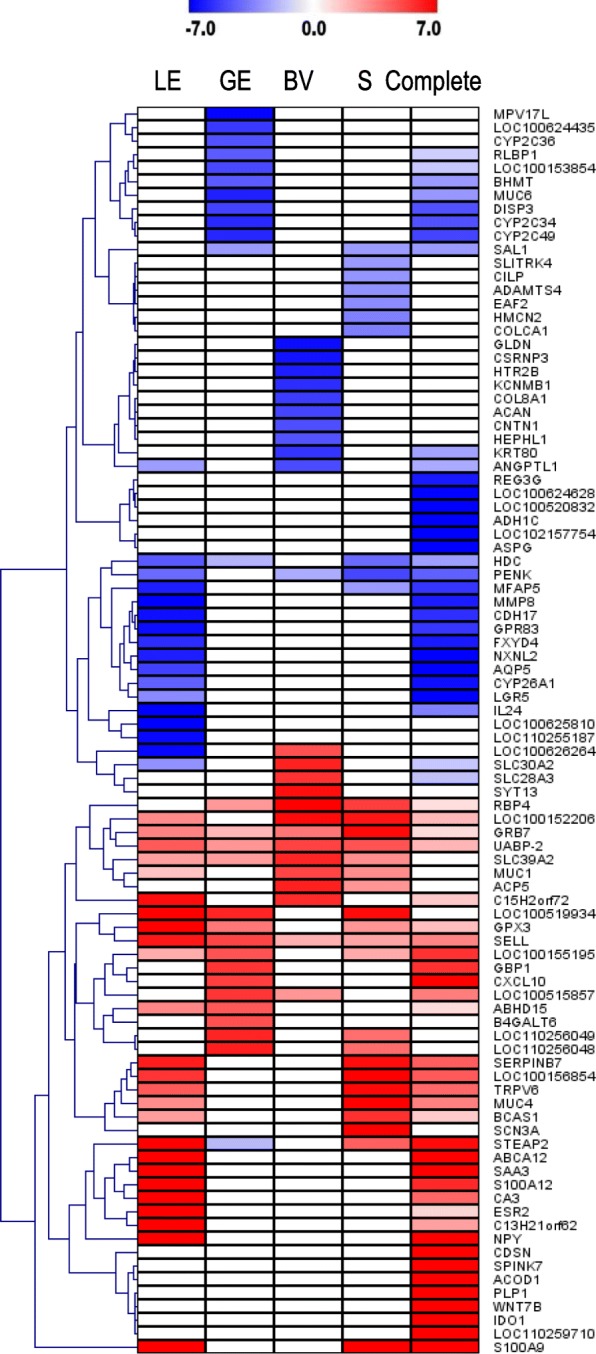
Heatmap of the top 10 upregulated and downregulated genes identified for each sample type in comparison of samples derived from pregnant and cyclic gilts. The color scale indicates log2 fold change from −7 to 7 (corresponding to a linear fold change of 128). Each column represents one LCM sample type or complete tissue (luminal epithelium (LE), glandular epithelium (GE), blood vessel (BV), stromal cells (S), and complete tissue). Detailed information can be found in Additional file 8: Table S5

The top 10 up- and downregulated genes of each sample type were selected to illustrate the very specific regulation of gene expression in endometrium on Day 14 of pregnancy (see Fig. 4). The genes, matrix metallopeptidase 8 (*MMP8*), cadherin 17 (*CDH17*), G protein-coupled receptor 83 (*GPR83*), FXYD domain containing ion transport regulator 4 (*FXYD4*), nucleoredoxin-like 2 (*NXNL2*), aquaporin 5 (*AQP5*), cytochrome P450, family 26, subfamily A, polypeptide 1 (*CYP26A1*), leucine rich repeat containing G protein-coupled receptor 5 (*LGR5*), interleukin 24 (*IL24*), olfactory receptor 6B3-like (*LOC100625810*) and uncharacterized *LOC110255187* were differential and only expressed in LE (Additional file 8: Table S5). Mitochondrial inner membrane protein like (*MPV17*), cytochrome P450 2C42-like (*LOC100624435*), cytochrome P450 2C36 (*CYP2C36*), retinaldehyde binding protein 1 (*RLBP1*), pancreatic alpha-amylase (*LOC100153854*), betaine-homocysteine S-methyltransferase (*BHMT*), mucin 6, oligomeric mucus/gel-forming (*MUC6*), dispatched RND transporter family member 3 (*DISP3*), cytochrome P450 2C34 (*CYP2C34*), cytochrome P450 2C49 (*CYP2C49*), guanylate binding protein 1, interferon-inducible (*GBP1*), C-X-C motif chemokine ligand 10 (*CXCL10*), and beta-1, 4-galactosyltransferase 6 (*B4GALT6*) were specifically differentially expressed in GE (Additional file 8: Table S5). Gliomedin (*GLDN*), cysteine and serine rich nuclear protein 3 (*CSRNP3*), 5-hydroxytryptamine receptor 2B (*HTR2B*), potassium calcium-activated channel subfamily M regulatory beta subunit 1 (*KCNMB1*), collagen type VIII alpha 1 chain (*COL8A1*), aggrecan (*ACAN*), contactin 1 (*CNTN1*), hephaestin like 1 (*HEPHL1*), keratin 80 (*KRT80*), and synaptotagmin 13 (*SYT13*) were identified as DEGs specifically in BV. In stroma, SLIT and NTRK like family member 4 (*SLITRK4*), cartilage intermediate layer protein (*CILP*), ADAM metallopeptidase with thrombospondin type 1 motif 4 (*ADAMTS4*), ELL associated factor 2 (*EAF2*), hemicentin 2 (*HMCN2*), colorectal cancer associated 1 (*COLCA1*), and sodium voltage-gated channel alpha subunit 3 (*SCN3A*) were differentially expressed. There were also some genes only detected in complete endometrial tissue, such as, regenerating islet-derived 3 gamma (*REG3G*), lithostathine-like (*LOC100624628* and *LOC100520832*), gamma polypeptide (*ADH1C*), small nuclear ribonucleoprotein F-like (*LOC102157754*), asparaginase (*ASPG*), corneodesmosin (*CDSN*), serine peptidase inhibitor, Kazal type 7 (*SPINK7*), aconitate decarboxylase 1 (*ACOD1*), proteolipid protein 1 (*PLP1*), Wnt family member 7B (*WNT7B*), indoleamine 2,3-dioxygenase 1 (*IDO1*), and membrane-spanning 4-domains subfamily A member 8-like (*LOC110259710*). Detailed information is shown in Additional file 8: Table S5.

### Cell type-specific DEGs

A number of cell type-specific DEGs (962, 439, 785, and 107) were obtained for LE, GE, BV, and S, respectively. These genes were only DE in one cell type or in one cell type and complete tissue (Fig. 1b). DAVID Functional Annotation Clustering was performed for these specific DEGs to identify overrepresented functional categories and pathways specific for each endometrial compartment. Furthermore, DEGs that were only identified in complete tissue were analyzed (Additional file 9: Table S6). Overrepresented functions including “regulation of cell death”, “intracellular signal transduction”, “cell migration”, “molecular function regulator”, “response to hormone”, “cellular protein modification process” and “cell morphogenesis involved in differentiation” were obtained for upregulated genes in LE. For the downregulated genes in LE the categories “cholesterol biosynthetic process” and “lipid biosynthetic process” were enriched. The upregulated genes in GE revealed high enrichment for “cellular response to chemical stimulus”, “cell migration”, “cell surface receptor signaling pathway”, “extracellular matrix organization”, and “vasculature development”. In contrast, “mitochondrial part”, “oxoacid metabolic process”, “coenzyme biosynthetic process”, and “cellular lipid metabolic process” were overrepresented for genes downregulated in GE. Enriched categories for genes with higher expression in BV derived from pregnant endometrium were involved in “anatomical structure formation involved in morphogenesis”, “embryonic morphogenesis”, “epithelium development”, “cell morphogenesis”, and “immune system development”, whereas functional categories “adherens junction”, “myofibril”, “cellular localization”, “actin filament-based process”, and “mitotic cell cycle” were found for the genes with decreased expression in BV. The most significant functional themes for the genes only found as upregulated in complete endometria were “immune response”, “response to cytokine”, “cell activation”, “response to external biotic stimulus”, “response to lipopolysaccharide”, and “programmed cell death”.

### DEGs involved in cell adhesion and modulation of immune response as potential main players in embryo implantation

Genes involved in cell adhesion and immune response signaling were identified to analyze their localization of differential expression. A selection of corresponding lists of genes was obtained from GO, KEGG pathways, Wiki Pathways, and previous studies (interferon-stimulated genes [31], synaptic adhesion-like molecules, focal adhesion, integrin cell surface interactions, integrin-mediated cell adhesion, cell junction organization, GO:0098609 cell-cell adhesion, interferon type I signaling pathways, Type II interferon signaling (IFNG), interferon alpha/beta signaling, interferon gamma signaling, IL-1 signaling pathway, structural pathway of interleukin 1 (IL-1), wnt signaling) and compared to the lists of DEGs for the LCM samples and complete tissue. In total, 407 DEGs related to these selected processes and pathways were assigned. With the LCM-RNA-seq, 97, 74, 91, and 44 were localized to LE, GE, BV, and S, respectively. The detailed information is shown in Additional file 10: Table S7. Most of the genes related to immune response were only found in complete tissue as DE and were upregulated. Two typical interferon-stimulated genes (ISGs), interferon alpha inducible protein 6 (*IFI6*) and interferon-induced transmembrane protein 3 (*IFITM3*) were downregulated in LE, whereas interferon regulatory factor 2 (*IRF2*) was upregulated. Typical ISGs were upregulated in complete tissue only, such as interferon induced protein, interferon-induced protein with tetratricopeptide repeats, interferon regulatory factor, MX dynamin like GTPase, poly (ADP-ribose) polymerase, signal transducer and activator of transcription, tripartite motif containing, and the ubiquitin specific peptidase families as well as ISG15 ubiquitin-like modifier and radical S-adenosyl methionine domain containing 2 (*RSAD2*). Genes related to cell adhesion processes were found as DE in all LCM samples and complete tissue samples. In LE, e.g., the integrin genes *ITGAM*, *ITGAV*, *ITGB3*, *ITGB5*, *ITGB6*, and selectin L (*SELL*) were found as upregulated. Overall, integrin genes showed complex expression patterns in the different endometrium compartments according to their complex and diverse functions. Likewise, members of the claudin family, important for cell junctions, showed complex patterns with *CLDN1* upregulated in LE, *CLDN22* downregulated in GE, *CLDN3*, 4 and 7 upregulated in BV, *CLDN11* upregulated in stroma, and *CLDN8* and 23 downregulated in complete tissue only.

## Discussion

The use of laser capture microdissection (LCM) to isolate samples derived from distinct endometrial compartments of the porcine endometrium for RNA-sequencing, provided a new insight into the regulation of the endometrial transcriptome during the preimplantation period. The results obtained for Day 14 of pregnancy revealed very specific gene expression and differential regulation in the studied endometrial compartments (luminal epithelium, LE; glandular epithelium, GE; blood vessels, BV; stroma, S). For example, the overlap of DEGs between the different compartments/cell types was much lower compared to our findings on Day 12 of pregnancy [25]. Compared to the analysis of complete endometrial tissue samples collected on Day 14 of pregnancy [22], differential gene expression was assigned to the functional compartments of the endometrium and a high number of genes (1822) was found as DE in LCM samples but not in the complete tissue samples. This number was also higher than the number of DEGs specifically found in LCM samples for Day 12 of pregnancy [25]. This is further supporting the results of our previous study [25] that the LCM approach provides cell-specific gene expression information that can be hidden in transcriptome analysis of whole endometrium tissue samples. Interestingly, there were also many DEGs (2119) only found for complete tissue samples but not detected as DE in LCM cells. This could result from the very low amount of starting material for the RNA-seq libraries in case of the LCM samples. However, given that the number of the detectable genes in the LCM samples was relatively high (between 11,000 and 12,000), this was probably the reason only for a part of those DEGs. Another reason could be that some cell types were not contained in the collected LCM samples but in the complete endometrial tissue samples. For LCM samples, LE, GE, larger blood vessels, and stromal areas without other visible structures were collected. Thus, for example immune cells located close to LE, GE, and smaller blood vessels were probably not present in LCM samples, but in complete endometrial biopsies. A number of DEGs, such as indoleamine 2, 3-dioxygenase 1 (*IDO1*), serine peptidase inhibitor, Kazal type 7 (putative) (*SPINK7*), and C-X-C motif chemokine ligand 9 (*CXCL9*), were strongly upregulated (log2 fold change 8.2, 9.8, 7.35, respectively) and only detectable in complete tissue. Indoleamine 2,3-dioxygenase 1 has been reported to play a role in suppressing T-cell activation in murine endometrium, and its mRNA expression is most likely localized to immune cells located around blood vessels [31]. In this study, the differential expression of *IDO1* was observed only in the complete endometrium samples that may be due to location in such immune cells. For *SPINK7*, expression in endometrium was not reported so far but in other tissues, a function in regulation of cell migration/invasion [32] and inflammatory responses [33] has been proposed. The chemokine *CXCL9* showed highest expression in porcine endometrium on Day 15 of pregnancy and expression has been primarily localized to stromal, endothelial, or vascular smooth muscle cells [34]. Results from cell migration assays suggested that CXCL9 may play a role in the recruitment of immune cells, such as T and NK cells into the endometrium during the implantation period in pigs [34]. These results showed that some endometrial cell populations, mainly immune cells are underrepresented in LCM samples collected from cresyl violet-stained frozen sections, indicating the importance of the analysis of the complete tissue sample as a control.

### Regulation of uterine luminal fluid secretion

Before the embryo completes the implantation, the uterine fluid is very critical for the embryo-maternal communication and nutrients for the embryo survival. Studies of the mechanism of uterine fluid secretion and reabsorption revealed that sodium channel epithelial 1 (SCNN1) and cystic fibrosis transmembrane conductance regulator (CFTR) play essential roles in the regulation of secretion [35]. In the present study, expression of *SCNN1A* was highest in LE, followed by GE, but only upregulated in BV and stroma at much lower expression levels compared to LE and GE. The *SCNN1* family members *SCNN1B* and *SCNN1G* were detected in LE and complete tissue but with lower expression in samples from pregnant animals, whereas *SCNN1D* was only detected in complete tissue and showed higher expression for the pregnant stage. In studies of *SCNNs* expression in the endometrium of the mouse, *SCNN1A* was mainly located on the apical membrane of both LE and GE [36], and the activation of *SCNN1* in the uterus was employed to initiate mouse embryo implantation [37]. In addition to the regulation of *SCNNs* in the uterus, serum/glucocorticoid regulated kinase 1 (*SGK1*), first found as a key factor of sodium transport regulation, has been proposed as an important regulator of reproductive success in mice and human [38]. Dysregulation of *SGK1* expression has been found in unexplained infertility and recurrent pregnancy loss in humans which was functionally characterized in mouse models [39]. In our study, *SGK1* mRNA was detected in all LCM samples as well as in complete tissue with highest expression in LE. Expression of *SGK1* was upregulated in complete tissue and in LE (adjusted *P*-value 0.02). Altogether, the complex pattern of *SCNN1* mRNA expression regulation together with members of the sodium voltage-gated channel family and *SGK1* shows fine-tuned regulation in the corresponding uterine compartment important for a positive pregnancy outcome. A previous study reported that cystic fibrosis transmembrane conductance regulator (*CFTR*) is abundantly expressed in stromal cells rather than the epithelial cells in mouse endometria [36]. In a cell culture model of porcine endometrial epithelial cells, the role of CFTR in Cl- secretion into the uterine lumen and regulation by PGE2 has been studied [40]. In the present study, *CFTR* mRNA was expressed in LE, GE (highest expression), and BV, but was not detectable in stromal areas. Interestingly, the expression of *CFTR* was upregulated in BV and downregulated in LE compared to Day 14 cyclic controls, resulting in no difference of *CFTR* expression in complete tissue. Overall, ion channels are playing an important role for endometrial receptivity and embryo/conceptus attachment by controlling the amount of uterine fluid [41]. Similar to humans and mice, the downregulation of *CFTR* in LE may contribute to reduced volume of uterine fluid in the pig. However, the expression patterns of mRNAs for SCNN1 channels are different compared to humans and mice, which could reflect the different developmental stage of the conceptus at the time of attachment to the uterine wall.

### The maternal-embryo cell-to-cell interaction

The processes of embryo migration and attachment are driven by several adhesion molecules, such as integrins, selectins, and cadherins which are located at the conceptus apposition and attachment sites. Differential expression of integrin genes including upregulation of *ITGAV*, *ITGA3*, *ITGB6*, and *ITGB8* in LE, and *ITGB6* also in GE was identified in our recent study on Day 12 [25]. Compared with the data from Day 12, the mRNAs for the integrin beta subunits associating with ITGAV, beta 1, 3, 5, 6 and 8 (https://www.ncbi.nlm.nih.gov/gene/3685) were all expressed in LE, and *ITGAV*, *ITGB3*, *ITGB5*, and *ITGB6* were upregulated (14-fold, 2.8-fold, 8.4-fold). Another integrin gene (*ITGAM*) was also found as upregulated in LE on Day 14. The highest expression in LE of the samples from Day 14 pregnant gilts (4-fold upregulated) among the integrin genes showed *ITGAV*. Other integrins with very high mRNA expression in LE were *ITGA2*, *ITGA6*, *ITGB1*, *ITGB4*, and *ITGB8*. However, they were not DE or were even downregulated (*ITGA6*). Integrin beta 3 and *ITGB5* were specifically upregulated in LE on Day 14, while there was no difference between the cyclic and pregnant LE on Day 12. In LE, the similar reads of *ITGB5* were found both in pregnant and nonpregnant pigs on Day 12 as well as on Day 14 of cyclic groups. However, the reads of *ITGB5* was dramatically increased on Day 14 of pregnancy. *ITGB6* showed the most specific expression, mainly in LE and only weak expression in GE. In GE, the *ITGB5* was upregulated on Day 14 instead of *ITGB6* compared with Day 12. Downregulation of *ITGB4* and *ITGA9* were identified in S on Day 12 and 14, respectively, and *ITGA3* was upregulated in S on Day 14. Interestingly, though the upregulation was found on Day 14, actually the reads of Day 14 was much lower than the data from Day 12, which suggest *ITGA3* transcripts were decreased along with the pregnant processing. In addition, upregulation of *ITGB8* was observed in BV, and 5 integrin genes (*ITGA3*, *ITGA7*, *ITGA9*, *ITGB1*, and *ITGBL1*) were downregulated in BV. The integrin heterodimers ITGAV/ITGB3 and ITGAV/ITGB6 have been shown to be involved in trophoblast attachment to the luminal epithelium in the pig [10]. Furthermore, ITGAV/ITGB3 and ITGAV/ITGB5 have been shown to mediate attachment of human trophoblast cells to endometrial epithelial cells in vitro [42]. The upregulation of *ITGB3* in LE could be attributed to regulation by Homeobox A10 (*HOXA10*) that was expressed in all LCM samples and upregulated in GE since it has been shown that *HOXA10* is able to induce *ITGB3* directly [43]. In addition, defective uterine receptivity in human endometrium is linked to decreased expression of *ITGAV* and *ITGB3* [44], suggesting that the upregulation of *ITGAV* and *ITGB3* in LE of porcine endometrium is important for embryo attachment. Overall, the complex regulation of the integrin genes on Day 14 in the endometrium, particularly in LE suggests a major role during initiation of embryo implantation.

In addition to the function of integrins in trophoblast to epithelium attachment, other roles during embryo implantation have been described. A leukocyte-specific integrin expressed on macrophages and NK cells has been identified to be formed from integrin ITGAM and ITGB2 (CD11b/CD18) [45]. In the goat, the number of CD11b positive cells, probably mature natural killer cells, increased in pregnant endometrium in response to the chemokine CXCL10 and were probably involved in creating an immune environment of the uterus suitable for conceptus implantation in ruminants [46]. In porcine endometrium, *CXCL10* mRNA was highly upregulated in complete pregnant endometrium but not in the LCM samples suggesting the *CXCL10* mRNA expression is located in immune cells not present in the LCM samples. Furthermore, expression of *ITGAM* mRNA was higher on Day 14 compared to Day 12 of pregnancy in porcine endometrium [25]. The finding that injection of an ITGAM antibody into the uterine lumen of early pregnant mice resulted in pregnancy loss further indicates an important role of this integrin during implantation [47]. The specific upregulation in LE and BV on Day 14 suggests expression in infiltrating immune cells such as regulatory NK cells that has to be proved in future studies.

The mRNA for L-selectin (*SELL*) was upregulated in all four LCM sample types as well as in complete tissue, but particularly in LE (84-fold) with high expression, suggesting that endometrial *SELL* could be involved in initiation of the embryo attachment process in the pig. Expression of SELL has been shown on trophoblasts of human blastocyst-stage embryos, while selectin oligosaccharide-based ligands were upregulated by uterine epithelium during the window of implantation in human [48]. Intriguingly, a related study on *SELL* with Holstein heifers showed that its mRNA and protein could be detected in the uterine epithelium but not in conceptuses during the periattachment period [49].

**Fig. 5:**
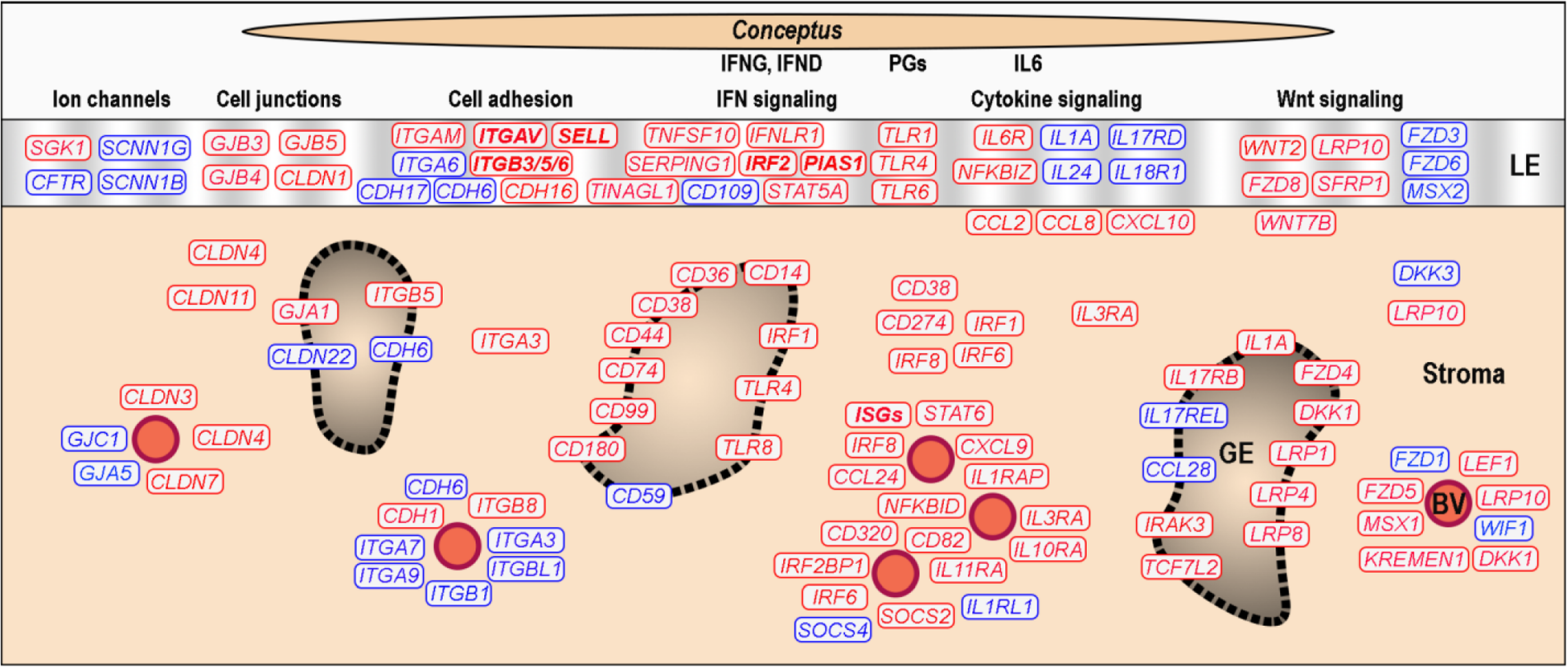
Summary of the main findings of the study. This schematic overview is based on the results of the present study of endometrial localization of differential gene expression. Genes highlighted in red and blue color were found as up- and downregulated, respectively when comparing pregnant to nonpregnant cyclic stage

Altogether, compared to our previous study of whole endometrial biopsies on Day 14 of pregnancy [22] the LCM RNA-seq approach significantly improved the interpretation of differential gene expression regarding the genes involved in conceptus attachment and implantation (Fig. 5).

### WNT signaling

WNT family members are considered as important factors involved in uterine developmental processes and implantation [50]. WNT signaling can be divided into the canonical and the non-canonical pathway regarding its specific functions [51]. The complex blastocyst-uterus interplay is connected to the WNT signaling pathway, and the canonical WNT signaling supports blastocyst competency for implantation [52]. WNT ligands can bind to frizzled (FZD) receptors and low density lipoprotein receptor-related protein (LRP) complex in order to transduce their signals [53]. In the present study, a number of members of the WNT signaling pathway were found as DE, such as *WNT*, *FZD, LRP, SFRP,* and *DKK* genes. WNT family members showed a very complex expression pattern in the endometrium, but only *WNT2* (upregulated in LE, downregulated in complete tissue) and *WNT7B* (310-fold upregulated in complete tissue, almost undetectable in cyclic stage and not found in LCM samples) were found as DE. In sheep, expression of *WNT2* was only found in stroma [54]. Expression of *WNT7B* has been found in human endometrium [55] and in the neonate mouse uterus [56]. In addition, WNT7B has been shown to be required for proper lung mesenchymal growth and vascular development [57]. The strong upregulation of *WNT7B* in porcine endometrium complete tissue samples may reveal a new role in the uterine preparation for implantation.

Like in sheep [54], upregulation of the Wnt signaling inhibitor *DKK1* was found in porcine endometrium. But in contrast to ovine endometrium, where *DKK1* mRNA increased in stroma from Day 16 of pregnancy, *DKK1* was upregulated on Day 14 of pregnancy in GE and BV and expressed in all endometrial compartments in the pig. Overall, upregulation of *DKK1* expression in the endometrium during the preimplatation period has been found in several species including human [3]. For another class of WNT signaling antagonist genes, the secreted frizzled related proteins (SFRPs), the genes encoding SFRP family members (*SFRP1*, *SFRP2*, and *SFRP4*), were detected in porcine endometrium. The localization of *SFRP1*, *SFRP2*, and *SFRP4* expression in the current study was in LE, S, and BV, respectively. There was no significant difference between the pregnant and cyclic group for *SFRP2* expression, but *SFRP1* was upregulated in LE (5.1-fold, adjusted *P*-value 0.011) and *SFRP4* was downregulated in complete tissue at a very low expression level. Apart from DKK and SFRP family proteins, WNT inhibitor factor (WIF) directly binds to WNT ligands [53]. WNT inhibitory factor 1 (*WIF1*) was lowly expressed in GE, BV and complete tissue, and it was identified as downregulated in BV. The binding of WIF1 with WNT occurs in the extracellular matrix and prevents the interaction between WNT and its receptor [58], and the low expressed WIF1 may support the cell communication via the extracellular matrix.

Of the frizzled class receptors, *FZD4* was upregulated in complete tissue, but the other genes (*FZD2*, *FZD3*, *FZD5*, *FZD6*, and *FZD7*) were all downregulated. With the LCM method, upregulation of *FZD8* and downregulation of *FZD3* and *FZD6* were found in LE. In sheep, expression of *FZD6* and *FZD8* was mainly found in endometrial epithelia during the periimplantation period [54]. *FZD4* and *FZD5* were upregulated in GE and BV, respectively. In addition, *FZD1* was downregulated in GE. In mouse and sheep endometria during the phase of gland development, *FZD2* and *FZD6* were detected in all uterine cell types, the latter particularly with abundant expression in endometrial epithelia [59]. Furthermore, other WNT receptors, *LRP8* and *LRP11* were upregulated, while *LRP12* was downregulated in complete tissue. More DE *LRP* genes were found in LCM samples, for instance, *LRP10* was upregulated in LE, BV, and stroma. *LRP1*, *LRP4*, and *LRP8* were found with significantly higher expression in GE, and *LRP2*, *LRP2BP*, and *LRPAP1* in BV of the pregnancy group. The dickkopf class of WNT signaling pathway inhibitors, especially DKK1, DKK2, DKK3, and DKK4 only bind to the LRP receptors and interrupt the canonical WNT signaling [60]. DKK inhibits WNT signaling by acting in concert with its receptor Kremen to form a ternary complex with LRP6 [60]. Here, *DKK1* was found as upregulated both in GE and BV and the gene encoding kringle containing transmembrane protein 1 (*KREMEN1*) was also found as expressed in all four LCM samples and as upregulated in BV (1.8-fold, adjusted *P*-value 0.009).

The important transcriptional regulators of WNT signaling pathway components, msh homeobox 1 and 2 (*MSX1* and *MSX2*) were both downregulated in LE and *MSX1* was upregulated in BV in the current study. The *MSX1* and *MSX2* mRNA were declined by P4 treatment in ovine uterus, which was supposed to alter tight and adherens junctions, thereby stimulating blastocyst growth and development [61]. In the mouse, *MSX1* was transiently expressed in LE and GE on Day 4 of pregnancy, but decreased with the onset of implantation (Day 5) to undetectable levels on Day 8 [62]. Deletion of both *Msx1* and *Msx2* leads to complete infertility and aberrant expression of implantation-related genes [63]. Additional transcriptional regulators of WNT signaling were found as DE in porcine endometrium, transcription factor 7 like 2 (*TCF7L2*) and lymphoid enhancer binding factor 1 (*LEF1*), upregulated in GE and in BV, respectively. In sheep, endometrial expression of TCF7L2 has been detected as transiently increased in epithelia of P4-treated ewes on Day 9, but decreased with longer P4 application on Day 12 [61]. From Days 10 to 20 of pregnancy in sheep, *LEF1* mRNA was detectable in LE and GE by in situ hybridization [54] whereas in the present study expression was detectable in all LCM samples derived from Day 14 of pregnancy.

Overall, members of the Wnt signaling pathway showed a very complex spatial expression pattern in porcine endometrium. Some of them showed even contrary regulation of expression in different endometrial compartments (see Fig. 5). Furthermore, the comparison to the findings in other species showed specific differences in temporal and spatial regulation, suggesting a specific role in the regulation of the type of epitheliochorial placentation in the pig. Wnt signaling is mediating interactions with the embryo, between different endometrial cells, angiogenesis, and maybe also with respect to regulation of infiltrating immune cells thereby playing an essential role in early pregnancy events [64].

### Cytokines and interferon regulatory factors

The interleukin family, i.e., a variety of cytokines and their receptors, is of great importance during embryo implantation [65]. A number of genes encoding interleukins and interleukin receptors was found as upregulated in LE on Day 12 of pregnancy in our previous study [25], such as, interleukin 1 receptor type 1 (*IL1R1*), interleukin 1 receptor accessory protein (IL1RAP), and interleukin 1 receptor associated kinases 3 and 4 (*IRAK3, IRAK4*). In contrast, only interleukin 6 receptor (*IL6R*) was upregulated on Day 14 in LE of pregnant gilts. The remaining DE interleukin family genes, interleukin 1 alpha and 24 (*IL1A*, *IL24*), interleukin 17 receptor D and 18 receptor 1 (*IL17RD*, *IL18R1*) were downregulated in LE of pregnancy. Interestingly, *IL24* expression was almost not detectable in LE of pregnant gilts (946-fold downregulation, only very low in one of 4 pregnant samples). Furthermore, *IL24* was not detectable in GE, BV and S. On Day 12, expression of *IL24* already started to decrease in samples from pregnant endometrium but was still expressed in all LCM samples [25]. In humans, IL-24 expression has been shown in villous and decidual tissues, trophoblasts, stroma and blood vessels during early pregnancy [66]. Furthermore, inhibition of invasiveness of a human trophoblast cell line was found in the same study. The downregulation of *IL24* on Day 14 in the pig could be related to trophoblast attachment to the endometrium. Expression of interleukin 6 (*IL6*) mRNA in the elongating porcine embryo has been described from Day 13 to Day 21 of pregnancy [67], and IL-6 activity has been detected in the uterine fluid during the preimplantation phase [68]. The 11-fold higher concentration of *IL6R* mRNA in pregnant compared to cyclic LE suggests that IL-6 and its receptor have also an important role in conceptus implantation in the pig as in other species such as human and mice [69].

Opposite regulation was observed for *IL1A*, upregulation in GE and downregulation in LE. In a recent study of the effects of PGF2a on porcine endometrium, the expression of IL1A was increased after treatment of endometrial explants with PGF2a [70]. In the context of a study with human cytotrophoblast cells, the proinflammatory cytokine IL-1A could also be involved in regulation of trophoblast invasiveness in the pig [71]. The importance of fine-tuned regulation of *IL1A* expression is indicated by a study in mice where IL-1A administration led to implantation failure [72]. In BV, upregulation of interleukin 3, 10 and 11 receptor subunit alpha (*IL3RA, IL10RA, and IL11RA*), and downregulation of interleukin 1 receptor like 1 (*IL1RL1*) were observed. Interleukin 10 (IL10) functions as a potent protector against vascular dysfunction, and enhancement of IL10 has been suggested as an immunotherapeutic intervention to treat adverse pregnancy outcomes [73]. The higher expression of *IL10RA* in BV of pregnant gilts suggested IL10 and IL10RA may play a role in vascular remodeling in normal pregnancy. Besides, upregulation of interleukin 1 receptor accessory protein (*IL1RAP*) and interleukin 3 receptor subunit alpha (*IL3RA*) were found in stroma in this study and a higher expression of IL1RAP in pregnant compared to cyclic endometrium has been shown in a related study [74]. Our results on Day 14 provided the additional information that *IL1RAP* was not only detected in LE and GE, but also in BV and S, and the upregulation of *IL1RAP* is mainly localized in BV of pregnant gilts (overview in Fig. 5).

Many genes of the chemokine system were found as expressed in the endometrium in this study. Interestingly, most of these genes, such as C-C motif chemokine ligands 2, 4, 5, 8, 26, (*CCL2*, *CCL4*, *CCL5*, *CCL8*, and *CCL26*), C-C motif chemokine receptor 1, 2, 3, 5, 7 (*CCR1*, *CCR2*, *CCR3*, *CCR5*, and *CCR7*), C-C motif chemokine receptor like 2 (*CCRL2*), C-X-C motif chemokine ligand 9, 11 (*CXCL9* and *CXCL11*), and C-X-C motif chemokine receptor 3 (*CXCR3*) were only DE in complete tissue, what could be because of expression in infiltrating immune cells located around smaller vessels or in subepithelial stromal areas. For example, expression of *CCL2* and *CCL8* (monocyte chemotactic protein-1 and -2) increased between Days 13 and 19 of pregnancy in the ovine uterus and was located in eosinophils recruited to the subepithelial compact stroma [75]. Another three chemokine members (*CCL3L1*, *CCL28*, and *CXCL10*) which were DE in complete endometrium were also DE in GE, whereas, *CCL24* was downregulated in complete endometrium and upregulated in BV. In agreement with the results of a previous study [76], CCL28 was mainly expressed in GE and at lower levels in samples from pregnant gilts. Messenger RNA expression of *CXCL9*, *CXCL10*, *CXCL11*, and *CXCR3* have been found highest on Day 15 of pregnancy in porcine endometrium [34]. In the same study, on Day 15 of pregnancy expression of *CXCL9* was localized to vascular endothelial cells, *CXCL10* exclusively to subepithelial stromal cells and endothelial cells, CXCL11 protein mainly in smooth muscle cells of BV, and CXCR3 protein primarily in vascular endothelial cells [34]. Han et al. [34] also showed that these chemokines are involved in the recruitment and migration of T cells and NK infiltrating the endometrium on Day 15 of pregnancy. In sheep, expression of CXCL10 (alias IP-10) has been shown in monocytes located in the subepithelial stroma of pregnant ewes [77]. A very recent study investigated a number of selected chemokines at the porcine maternal-fetal interface during the periimplantation period and revealed CCL2, CCL5, CCL11 and CXCL12 as involved in communication with the trophoblast, and suggested that CXCL9 and CXCL10 are involved in recruitment of immune cells and establishment of an immunotolerant environment for conceptus implantation [78]. Overall, chemokines are supposed to be involved in conceptus development, lymphocyte-promoted endometrial angiogenesis important for conceptus survival, and in pregnancy success in general [79, 80]. Although the importance of the chemokine system for establishment and maintenance of pregnancy in the pig has been shown, our study provides in addition the complexity of gene expression regulation and location of expression of the corresponding genes in porcine endometrium on Day 14 of pregnancy.

Similar to ruminants, transcriptional repressor interferon regulatory factor 2 (*IRF2*) has been found as upregulated in the endometrial LE from Day 12 of pregnancy in the pig thereby limiting upregulation of interferon-stimulated genes (ISGs) to glandular and stromal regions [25, 81, 82]. In contrast to ruminants, where the conceptus secretes the type I IFN IFN tau [83, 84], the porcine conceptus is secreting type II IFNs (IFN gamma (IFNG)) and type I IFNs (IFN delta (IFND)) during the periimplantation period [85, 86]. Previously, we found that the expression of ISGs was DE in porcine endometrium on Day 12 of pregnancy including upregulation of *IRF2* in LE [25]. In the present study of Day 14, many ISGs genes were found as DE, e.g., several genes encoding interferon-regulatory factors (*IRF1*, *IRF2*, *IRF4*, *IRF5*, *IRF6*, *IRF7*, and *IRF8*). The gene *IRF1* was identified as upregulated in GE and stroma, *IRF6* and *IRF8* in BV and stroma, and *IRF4* and *IRF7* only in complete endometrial tissue samples. The results for *IRF1* and the upregulation of *IRF2* in LE on Days 12 and 14 are consistent with previous studies [82] whereas the other IRFs have not been described so far in porcine endometrium.

Another *ISG*, signal transducer and activator of transcription 2 (*STAT2*), has been found with increased expression during the peri-implantation period compared with nonpregnant sows mainly localized in stratum compactum stroma [82]. In our study, we did not only confirm the upregulation of *STAT2*, but also identified more members of the STAT gene family (*STAT1*, *STAT4*, and *STAT5A*) as upregulated in complete endometrial tissue. Besides, upregulation of *STAT5A*, *STAT5B* was also found in LE, and *STAT6* was upregulated in BV. The upregulation of *STAT5A* in LE may be induced by estrogen as it has been shown in the mouse [87]. Many more of the typical ISGs [31, 88], such as *ISG15*, *ISG20*, *IFI44*, *IFIT1*, *MX1*, *MX2*, *USP18*, *GBP1–6*, *PARP* family members etc. were found as upregulated in the present study but mainly in complete tissues only or in BV and/or stroma.

Interestingly, a number of ISGs (*C1R*, *C1S*, *C3*, *C4A*, *DDX52*, *DHX34*, *FAM13A*, *GBP4*, *IFI27L2*, *IFI30*, *IRF2*, *JAK1*, *PIAS1*, *SERPING1*, *STAT5A*, *TINAGL1*, *TNFSF10*, *UBE2B*) were identified as upregulated in LE. The function in the endometrium of some of these genes has been described in other species, such as for tubulointerstitial nephritis antigen-like 1 (*TINAGL1*) in mice, where it is markedly expressed in postimplantation decidual endometrium and interacting with integrins [89]. The mRNA for *SERPING1*, encoding a regulator of complement activation, has been found as upregulated in bovine endometrium during the preimplantation period [90] and with decreased expression in endometrial biopsies collected on Day LH + 7 (window of implantation, WOI) from women with recurrent miscarriages [91]. Another gene that has probably a conserved function in different mammalian species is tumor necrosis factor (ligand) superfamily member 10 (*TNFSF10*). The *TNFSF10* mRNA has been found as upregulated in human endometrium during the WOI [92], in bovine endometrium on Day 18 of pregnancy [93], and in equine endometrium on Day 12 of pregnancy [94]. Protein inhibitor of activated STAT 1 (PIAS1) has been shown to block IRF3 DNA-binding activity and thereby negatively modulating type I IFN signaling [95], which is suggesting PIAS1 as another factor involved in repression of ISG expression in LE.

Porcine MHC class I (*SLA-1* to *8*) and class II (*SLA-D*) genes were differentially expressed in the endometrial tissue samples on Day 14, i.e., identified as upregulated in complete tissue samples derived from pregnant gilts. For the LCM samples, high expression was found for *SLA-1* to *3* in LE, BV, stroma, and moderate expression in GE. Upregulation of these MHC class I genes was only found in LE (log2 FC 2 to 3). The expression of most of the MHC class II genes (*SLA-DMA, SLA-DMB, SLA-DOA, SLA-DOB, SLA-DQA1, SLA-DQB1, SLA-DRA, SLA-DRB1, LOC100155975, LOC100153139, LOC106504372*) was low or absent in the LCM samples, particularly in LE and GE. Only *SLA-DQA1, SLA-DQB1, SLA-DRA, SLA-DRB1* showed moderate expression in BV and stroma, but no difference between pregnant and cyclic samples. In contrast, expression of class II genes was much higher and upregulated in complete tissue samples, indicating localization in immune cells present in areas not collected by LCM. This is in agreement with the results of a previous study, where *SLA-DQA, SLA-DQB* have been found as upregulated on Day 15 of pregnancy and mRNA and protein expression was detected in subepithelial stromal cells and around BV [96]. These are probably areas not contained in the LCM samples collected in our study but in the complete tissue samples. In another study, expression of the classical MHC class I genes *SLA-1*, *SLA-2*, and *SLA-3*, and the nonclassical class I genes *SLA-6*, *SLA-7*, and *SLA-8*, was studied in porcine endometrium during cycle and pregnancy [97]. Expression during pregnancy increased until Day 14 and decreased thereafter. Localization by in situ hybridization revealed expression of all SLA genes in LE, GE, and BV until Day 12 of the cycle and pregnancy, whereas expression decreased in LE from Day 15 and was not detectable from Day 20 on [97]. Since we found the classical SLA class I genes still with high expression in LE and upregulated in pregnant samples on Day 14, the downregulation in LE seems to start after Day 14 of pregnancy. This is also in agreement with a study in sheep, where MHC class I and beta2-microglobulin was absent in LE and superficial ductal GE, presumably caused by IRF2 upregulation in LE induced by IFNT [98]. Since upregulation of *IRF2* was also specifically found in LE in the present study and our recent study of Day 12 of pregnancy [25], the regulation of MHC gene expression could also be via conceptus interferons. Collectively, a complex spatial regulation of genes of various cytokine signaling systems is needed for the modulation of the immune system in preparation of conceptus attachment and implantation that is controlled by various signaling molecules secreted by the conceptus such as interferons, interleukins and chemokines [99].

## Conclusions

Using an integrated LCM and transcriptomic approach, the present study has revealed spatial information for differential gene expression in the porcine endometrium during the conceptus attachment phase. This significantly increased the depth of gene expression analysis results obtained in our recent study of porcine endometrium on Day 14 of pregnancy [22] and uncovered local differential gene expression hidden in the analysis of complete endometrial tissue samples. The assignment of differential gene expression to functional compartments of the endometrium provided an improved view on how biological processes involved in conceptus implantation could be regulated at this stage, such as control of uterine fluid secretion, trophoblast to endometrium adhesion, growth regulation by Wnt signaling, and modulation of the maternal immune system. The obtained results showed that an even higher spatial resolution with respect to specific regions of endometrial compartments, such as subepithelial stromal regions or even individual endometrial cell types, e.g., immune cells is needed to fully understand the complexity of regulatory processes in the context of establishment of pregnancy.

## Methods

### Target cell collection

The animal trial and uterus sample collection were conducted as described in our previous study [22]. Treatments of gilts were performed in accordance with the local authorities (District Government of Upper Bavaria). The performed standard procedures/treatments in animal breeding all followed the International Guiding Principles for Biomedical Research Involving Animals. Briefly, a number of 8 prepuberal gilts were synchronized with 750 IU eCG (Intergonan, MSD Animal Health Innovation GmbH, Schwabenheim, Germany), followed by 750 IU hCG (Ovogest, MSD Animal Health Innovation) after 72 h. “Pregnant” gilts (*n* = 4) were inseminated with a standard dose of German Landrace semen twice (24 h and 36 h after hCG injection), and “non-pregnant” (n = 4) were inseminated with the supernatant semen (3000 rpm, 10 min) from the same boar. The animals were slaughtered on Day 14 after insemination at the slaughterhouse of the Bavarian State Research Center for Agriculture, Grub, Germany. The animals were rendered unconscious by electrical stunning and then immediately bled by cutting the throat. The uteri were removed, and each uterine horn was subsequently opened longitudinally at the antimesometrial side and the hyperemic zones (the sites of embryonic attachment) were visible in the pregnant endometrium. In the pregnant sows, endometrial samples (including the lamina epithelialis, lamina propria, and tela submucosa but not tunica muscularis) were collected from the hyperemic zones after carefully removing the conceptus. Then, the endometrial tissue samples were frozen immediately in liquid nitrogen and stored in − 80° for further analysis. Using a clinical cryostat (Leica CM1950, Leica Biosystems, Germany), 10 μm thick sections of endometrial tissue were cut to mount on membrane slides (MembraneSlide NF 1.0 PEN, Zeiss, Germany), followed by a modified staining protocol. All solutions used for staining were prepared with RNase-free water. Briefly, the slides were in 70% ethanol for fixation, 50% ethanol for washing, and 1% cresyl violet for staining. After staining, the sections were washed by 50, 70, and 100% ethanol, respectively, and dried in room temperature for 3 mins. Finally, isolation of target cell was performed on PALM Microbeam (Zeiss PALM Microsystems, Germany) to identify LE, GE, BV, and stromal cells. The LCM cells were collected with the LCM cap (AdhesiveCap 200 clear, Zeiss, Germany) and incubated with 50 μl extraction buffer at 42 °C for 30 min to lyse the cells. The targeted cell types after staining were visible in the endometrial tissue with PALM Microbeam (see Additional file 3: Figure S6 for LE, GE, BV, and stroma), and the collected cells are shown in Additional file 3: Figure S7.

### RNA extraction and library preparation

PicoPure RNA Isolation Kit (Applied Biosystems™, Vilnius, Lithuania) was used to extract the total RNA from isolated LE, GE, BV, and stromal cells of individual pig following the manufacturers’ instructions. After RNA isolation, each RNA sample was performed on the Agilent 2100 Bioanalyzer (Agilent Technologies, Waldbronn, Germany) with the Agilent RNA 6000 Pico assay to assess RNA integrity and quantity. RNA Integrity number (RIN) of all samples ranged from 6.1 to 8.7, and most samples’ RIN number were around 7.5. Total RNA with 800 pg input was used for starting the library preparation, then a number of 32 RNA samples with 4 biological replicates in each cell type were prepared following the Ovation SoLo Single Cell RNA-Seq System (NuGen Technologies, San Carlos, USA). It was worth to notice that the number of PCR cycles was set with 16 during the amplification. Finally, a total number of 32 individual libraries with unique barcodes were mixed within three pools for one lane sequencing with single-read flow cell on an Illumina HiSeq 2500 instrument. The process of sequencing and demultiplexing was provided by the Functional Genomics Center Zurich (FGCZ).

### Bioinformatics analysis

The RNA-seq data analysis was conducted on our local Galaxy installation [100]. Briefly, the raw reads were subjected quality control checking firstly, then the adaptor was trimmed and 5 bp from 5′ end of the read was removed using Trim Galore. All the fragmented reads were mapped to the reference genome (Sscrofa 11.1) from NCBI (ftp://ftp.ncbi.nih.gov/genomes/Sus_scrofa/GFF) by using Hisat2 tool, and duplicates generated from the PCR amplification were cleaned with NUGEN nudup. The reads for each gene were quantified with the QuasR qCount tool. After that, the read count table was subjected to CPM cut-off filtering to remove genes with neglectable read counts. Genes passing this filter were defined as “detectable genes”. Statistical analysis of the read count data was performed in EdgeR (using GLM_robust) to identify DEGs [29]. In addition, DESeq2 [30] was used to confirm that similar results are obtained with both methods. An FDR of 1% in LE was set as cut-off and the corresponding *P*-value was used as the cut-off for the remaining three cell types in order to optimally compare the results. Then, these DEGs were subjected to hierarchical cluster analysis in MultiExperiment Viewer (MeV) for each cell type. The functional classification and pathway analyses related to these DEGs in each cell type were performed with Database for Annotation, Visualization, and Integrated Discovery (DAVID) [101]. Data analysis of complete endometrial tissue samples followed the same workflow except the step of removal of PCR duplicates. Raw FASTQ files used in current study were deposited at National Center for Biotechnology Information (NCBI) Gene Expression Omnibus (GSE123265).

## Supplementary information

**Additional file 1:** **Figure S1.** The expression profiles of DEGs between pregnant and nonpregnant endometrium for luminal epithelium (LE). **Figure S2.** The expression profiles of DEGs between pregnant and nonpregnant endometrium for glandular epithelium (GE). **Figure S3.** The expression profiles of DEGs between pregnant and nonpregnant endometrium for blood vessel (BV). **Figure S4.** The expression profiles of DEGs between pregnant and nonpregnant endometrium for stromal cells (S).

**Additional file 2:** **Figure S5.** Venn diagram showing the overlap of DEG for complete tissue samples obtained by EdgeR and DESeq2 (for both FDR cut-off 1%).

**Additional file 3:** **Figure S6.** Endometrial tissue sections after staining. The localization of luminal epithelium (LE), glandular epithelium (GE), blood vessel (BV), and stromal cells (S) in the endometrium. **Fig. S7.** Collected target cell areas. Luminal epithelium (LE), glandular epithelium (GE), blood vessel (BV), and stromal cells (S) in the endometrium were isolated by laser capture microdissection.

**Additional file 4:** **Table S1.** Raw data statistics of RNA-seq results.

**Additional file 5:** **Table S2.** Detectable genes in luminal epithelium (LE), glandular epithelium (GE), stroma (S), and complete tissue.

**Additional file 6:** **Table S3.** Differentially expressed genes in luminal epithelium (LE), glandular epithelium (GE), stroma (S), and complete tissue.

**Additional file 7:** **Table S4.** Comparative DAVID GO chart analysis.

**Additional file 8:** **Table S5.** Top 10 up- and downregulated genes in luminal epithelium (LE), glandular epithelium (GE), blood vessels (BV), stromal cells (S), and complete tissue.

**Additional file 9:** **Table S6.** Overrepresentation analysis for genes only differentially expressed in three cell types and complete tissue.

**Additional file 10:** **Table S7.** DEGs involved in cell adhesion and immune response signaling pathways.

## Data Availability

All data used in this study have been included in the article and its supplementary files. The sequence data (https://www.ncbi.nlm.nih.gov/geo/query/acc.cgi?acc=GSE123265) is available at National Center for Biotechnology Information (NCBI) Gene Expression Omnibus (GSE123265).

## Abbreviations

ACAN: Aggrecan
ACKR: Atypical chemokine receptors
ACOD1: Aconitate decarboxylase 1
ADAMTS4: ADAM metallopeptidase with thrombospondin type 1 motif 4
ADH1C: Gamma polypeptide
AQP5: Aquaporin 5
ASPG: Asparaginase
B4GALT6: beta-1, 4-galactosyltransferase 6
BHMT: Betaine-homocysteine S-methyltransferase
BV: Blood vessel
CCL: C-C motif chemokine ligand
CCL2: C-C motif chemokine ligand 2
CCR1: C-C motif chemokine receptor 1
CCRL2: C-C motif chemokine receptor like 2
CDH1: Cadherin 1
CDH17: Cadherin 17
CDSN: corneodesmosin
CFTR: Cystic fibrosis transmembrane conductance regulator
CILP: Cartilage intermediate layer protein
CNTN1: Contactin 1
COL8A1: Collagen type VIII alpha 1 chain
COLCA1: Colorectal cancer associated 1
CSRNP3: Cysteine and serine rich nuclear protein 3
CXCL: C-X-C motif chemokine ligand
CXCR3: C-X-C motif chemokine receptor 3
CYP26A1: Cytochrome P450, family 26, subfamily A, polypeptide 1
CYP2C34: Cytochrome P450 2C34
CYP2C36: cytochrome P450 2C36
CYP2C49: Cytochrome P450 2C49
DEGs: Differentially expressed genes
DISP3: Dispatched RND transporter family member 3
DKK: Dickkopf WNT signaling pathway inhibitors
E2: Estrogen
EAF2: ELL associated factor 2
ECM: Extracellular matrix
FXYD4: FXYD domain containing ion transport regulator 4
FZD: Frizzled
GBP1: Guanylate binding protein 1, interferon-inducible
GE: Glandular epithelium
GLDN: Gliomedin
GO: Gene Ontology
GPR83: G protein-coupled receptor 83
HEPHL1: Hephaestin like 1
HMCN2: Hemicentin 2
HTR2B: 5-hydroxytryptamine receptor 2B
IDO1: Indoleamine 2, 3-dioxygenase 1
IFI6: Interferon alpha inducible protein 6
IFITM3: Interferon-induced transmembrane protein 3
IFNs: Interferons
IL17RD: Interleukin 17 receptor D
IL18R1: Interleukin 18 receptor 1
IL1B2: Interleukin 1 beta 2
IL1R1: Interleukin 1 receptor type 1
IL24: Interleukin 24
IRAK: Interleukin 1 receptor associated kinases
IRF2: Interferon regulatory factor 2
ISGs: Interferon-stimulated genes
ITGs: Integrins
KCNMB1: Potassium calcium-activated channel subfamily M regulatory beta subunit 1
KREMEN1: Kringle containing transmembrane protein 1
KRT80: Keratin 80
LCM: Laser capture microdissection
LE: Luminal epithelium
LGR5: G protein-coupled receptor 5
LRP: Lipoprotein receptor-related protein
MMP8: Matrix metallopeptidase 8
MPV17: Mitochondrial inner membrane protein like
MSX: Msh homeobox
MUC6: Mucin 6, oligomeric mucus/gel-forming
NXNL2: Nucleoredoxin-like 2
OPN: Osteopontin
PLP1: Proteolipid protein 1
REG3G: Regenerating islet-derived 3 gamma
RLBP1: Retinaldehyde binding protein 1
RSAD2: Radical S-adenosyl methionine domain containing 2
S: Stromal areas
SCN3A: Sodium voltage-gated channel alpha subunit 3
SCNN1: Sodium channel epithelial 1
SELL: Selectin L
SFRP: Secreted frizzled related protein
SGK1: Serum/glucocorticoid regulated kinase 1
SLITRK4: SLIT and NTRK like family member 4
SPINK7: Serine peptidase inhibitor, Kazal type 7
STAT: Signal transducer and activator of transcription factors
SYT13: Synaptotagmin 13
WIF1: WNT inhibitory factor 1
WNT7B: Wnt family member 7B
