# Supplementary figures and images for: Spatial organization of endometrial gene expression at the onset of embryo attachment in pigs
Source: BMC Genomics. 2019 Nov 21;20:895. doi: 10.1186/s12864-019-6264-2 (PMC6873571; doi:10.1186/s12864-019-6264-2)

**
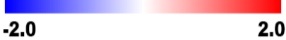
**

**Fig. S1**

**Fig. S2**

**
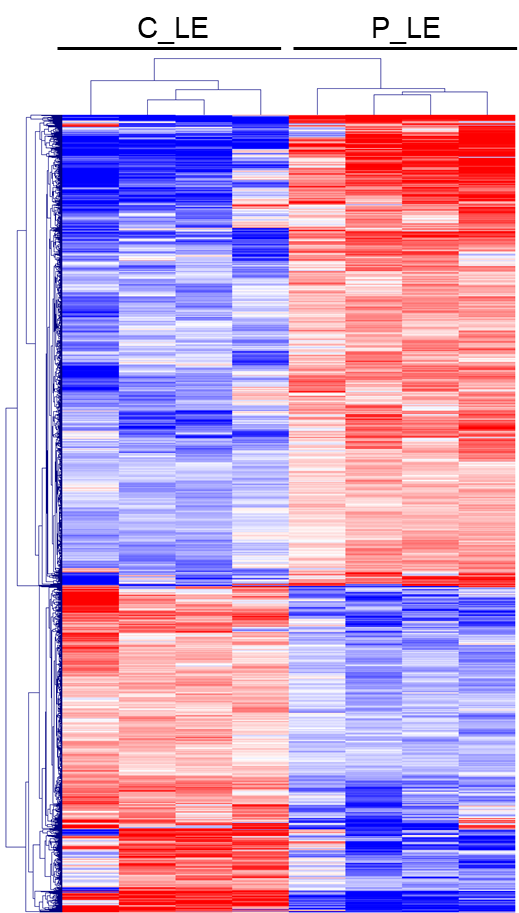
**

**
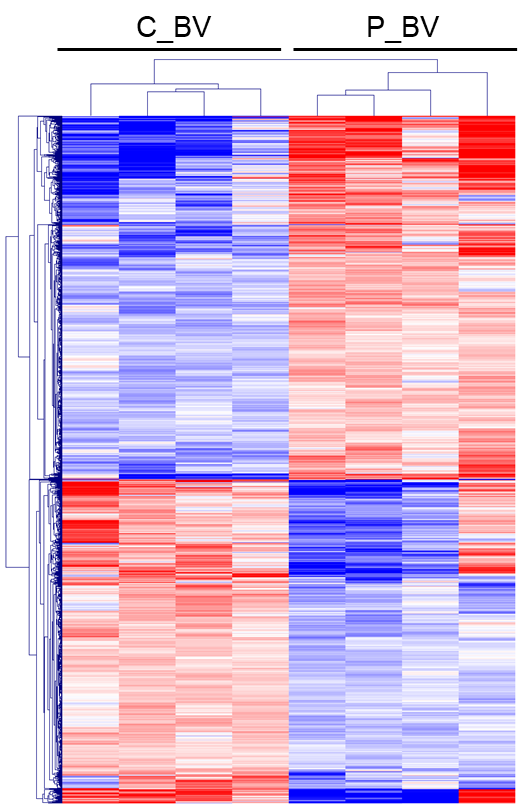
**

**Fig. S3**

**
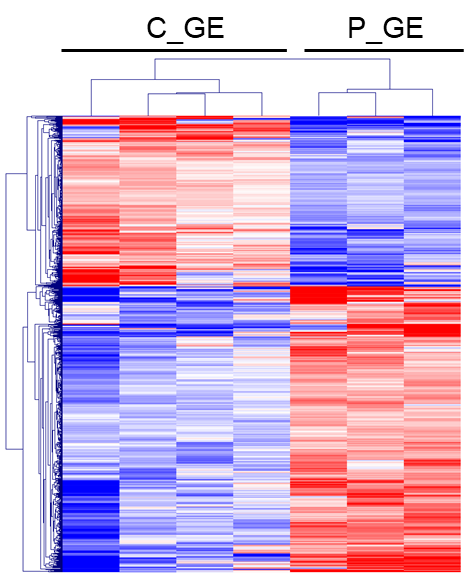
**

**Fig. S4**

**
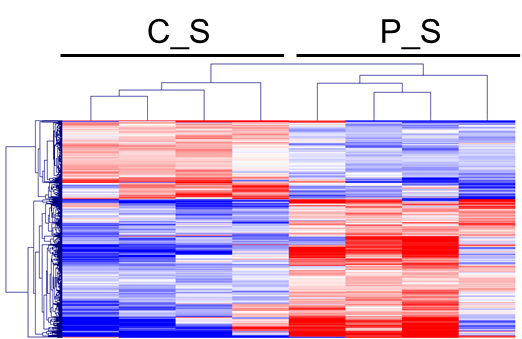
**

Supplement: Supplementary file 1 — Additional file 1: Figure S1. The expression profiles of DEGs between pregnant and nonpregnant endometrium for luminal epithelium (LE). Figure S2. The expression profiles of DEGs between pregnant and nonpregnant endometrium for glandular epithelium (GE). Figure S3. The expression profiles of DEGs between pregnant and nonpregnant endometrium for blood vessel (BV). Figure S4. The expression profiles of DEGs between pregnant and nonpregnant endometrium for stromal cells (S). [file 12864_2019_6264_MOESM1_ESM.docx]

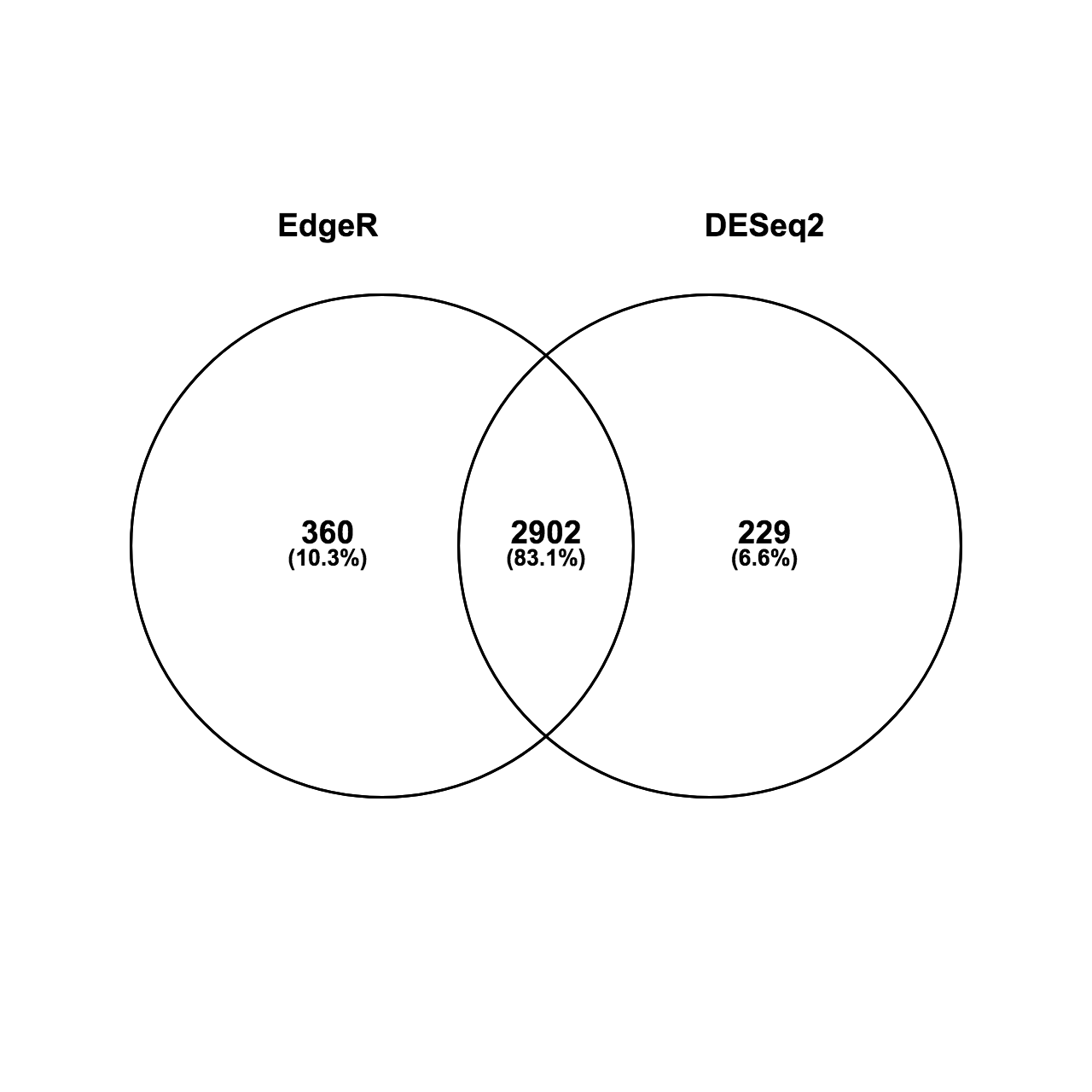

Supplement: Supplementary file 2 — Additional file 2: Figure S5. Venn diagram showing the overlap of DEG for complete tissue samples obtained by EdgeR and DESeq2 (for both FDR cut-off 1%). [file 12864_2019_6264_MOESM2_ESM.png]

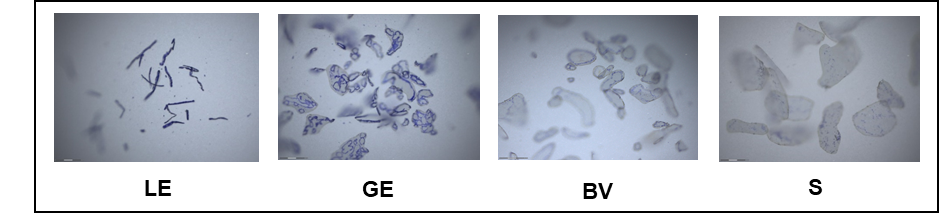


**Fig. S6**


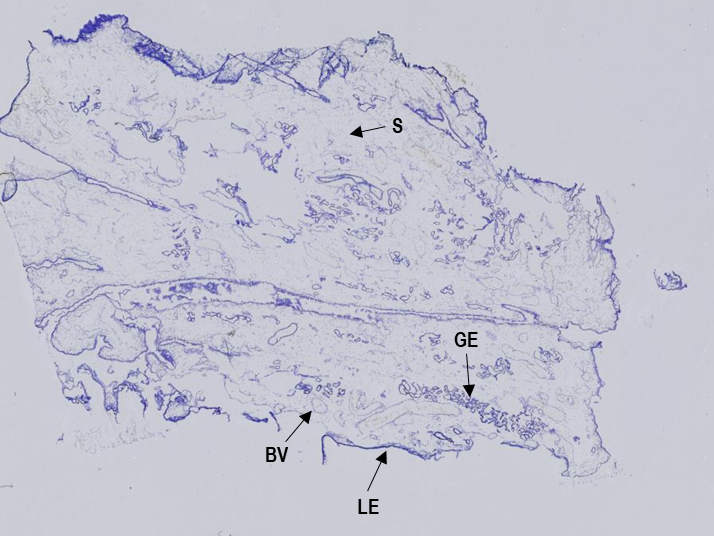


**Fig. S7**

Supplement: Supplementary file 3 — Additional file 3: Figure S6. Endometrial tissue sections after staining. The localization of luminal epithelium (LE), glandular epithelium (GE), blood vessel (BV), and stromal cells (S) in the endometrium. Fig. S7. Collected target cell areas. Luminal epithelium (LE), glandular epithelium (GE), blood vessel (BV), and stromal cells (S) in the endometrium were isolated by laser capture microdissection. [file 12864_2019_6264_MOESM3_ESM.docx]
