# Supplementary material for: Spatial organization of endometrial gene expression at the onset of embryo attachment in pigs
Source: BMC Genomics. 2019 Nov 21;20:895. doi: 10.1186/s12864-019-6264-2 (PMC6873571; doi:10.1186/s12864-019-6264-2)
Supplement: Supplementary file 4 — Additional file 4: Table S1. Raw data statistics of RNA-seq results. [file 12864_2019_6264_MOESM4_ESM.docx]

**Supplemental Table 1. Raw data statistic of RNA-seq**

| **Sample** | **Total Raw Reads** | **Total clean reads** |
| --- | --- | --- |
| C_B_128 | 12004844 | 11449670 |
| C_B_40 | 13619302 | 12514443 |
| C_B_42 | 13528609 | 12546372 |
| C_B_78 | 11331377 | 10431436 |
| C_G_128 | 15051382 | 14411973 |
| C_G_40 | 13151950 | 12246408 |
| C_G_42 | 14623638 | 13728883 |
| C_G_78 | 13459451 | 12540248 |
| C_L_128 | 12698634 | 12110032 |
| C_L_40 | 15756074 | 14619060 |
| C_L_42 | 12556050 | 11698188 |
| C_L_78 | 27691996 | 25612739 |
| C_S_128 | 22658617 | 21630769 |
| C_S_40 | 16826627 | 15682053 |
| C_S_42 | 12968552 | 12079676 |
| C_S_78 | 21270840 | 20296318 |
| P_B_139 | 12852259 | 12256022 |
| P_B_39 | 11408334 | 10889751 |
| P_B_41 | 12475997 | 11933414 |
| P_B_43 | 14916279 | 14261500 |
| P_G_139 | 22277483 | 21237876 |
| P_G_39 | 13174795 | 12639050 |
| P_G_41 | 12724471 | 12161594 |
| P_G_43 | 12707081 | 12132154 |
| P_L_139 | 21437642 | 20497050 |
| P_L_39 | 11195789 | 10714424 |
| P_L_41 | 26833543 | 25625442 |
| P_L_43 | 11363678 | 10872213 |
| P_S_139 | 18989205 | 18142040 |
| P_S_39 | 13335458 | 12709923 |
| P_S_41 | 13437739 | 12839471 |
| P_S_43 | 21833689 | 20772617 |
